# Association of free-water imaging data for the cholinergic nucleus with the motor function and subtypes in Parkinson’s disease

**DOI:** 10.3389/fneur.2025.1477827

**Published:** 2025-04-04

**Authors:** Shanhu Xu, Xiaoli Si, Miao Cai, Fengli Fu, Jun Tian, Baorong Zhang, Xiaoli Liu

**Affiliations:** ^1^Department of Neurology, Affiliated Zhejiang Hospital Zhejiang University School of Medicine, Hangzhou, China; ^2^Department of Neurology, The Second Affiliated Hospital Zhejiang University School of Medicine, Hangzhou, China; ^3^Department of Radiology, Affiliated Zhejiang Hospital Zhejiang University School of Medicine, Hangzhou, China

**Keywords:** Parkinson’s disease, motor subtypes, free-water imaging, cholinergic nucleus 4, pedunculopontine nucleus

## Abstract

**Background:**

Despite the importance of clinical heterogeneity in Parkinson’s disease (PD), its underlying pathophysiology remains unclear.

**Objective:**

This study aimed to distinguish the association of free-water (FW) imaging data for the cholinergic nuclei with the motor subtypes of PD.

**Methods:**

The study included 150 cases of idiopathic PD from the Parkinson’s Progression Markers Initiative cohort. FW imaging, including FW-corrected diffusion tensor imaging, was used to extract structural metrics from cholinergic nucleus 4 (Ch4) in the basal forebrain and the pedunculopontine nucleus. The motor subtypes were classified as tremor-dominant (TD, *n* = 99) and non-tremor-dominant (non-TD, *n* = 51). Statistical analyses were performed at baseline and the 4-year follow-up.

**Results:**

At baseline, FW value for Ch4 (FW-Ch4) was correlated with the tremor subscore, while FW-corrected fractional anisotropy in Ch4 (FA-t-Ch4) was negatively correlated with the rigidity subscore. However, the TD and non-TD groups showed no differences in cholinergic FW imaging data. Among the 84 patients who were followed-up, 36.36% (20/55) in the TD group and 34.48% (10/29) in the non-TD group showed a subtype shift after 4 years. Multivariate binary logistic regression analysis showed that the normalized FW value for Ch4 (nFW-Ch4) was a predictor of subtype at the 4-year follow-up (*p* = 0.041). In the TD subgroup, both nFW-Ch4 (*p* = 0.015) and normalized FW-corrected mean diffusivity in Ch4 (MD-t-Ch4) (*p* = 0.013) predicted subtype stability. The area under the receiver operating characteristic curve values were 0.69 and 0.73, respectively.

**Conclusion:**

Tremor and rigidity subscores were correlated with Ch4 FW imaging data. Moreover, Ch4 FW imaging predicted the motor subtype at the 4-year follow-up, especially identifying potential postural instability and gait difficulty (PIGD) subtype converters from the TD group.

## Introduction

Parkinson’s disease (PD) is a progressive neurodegenerative disease characterized by heterogeneous clinical and neuropathological manifestations. The most common manifestations include the classic triad of motor symptoms, namely, resting tremor, rigidity, and bradykinesia and postural instability. Establishment of different subcategories is an important priority in clinical research on PD (1), since these subtypes have implications for diagnosis, prognosis, and expected treatment responses (2, 3). Although different algorithms to determine PD motor subtypes have been proposed, the subtypes based on Jankovic’s classification system, which categorizes patients into tremor-dominant (TD), indeterminate (ID), and postural instability and gait difficulty (PIGD) subtypes on the basis of motor dominance, are most frequently studied in the literature (4–6) and more suitable for identifying non-motor abnormalities (6). Nevertheless, due to the instability of motor subtypes, the Parkinson’s Progression Markers Initiative (PPMI) consortium decided to categorize patients with PD into TD and non-TD (PIGD and ID) subtypes (7).

In addition to experiencing motor symptoms, patients with PD also show non-motor symptoms (NMS) such as sleep disturbances, autonomic dysfunction, cognitive decline, and psychiatric symptoms. NMS may even appear earlier than the classic motor features in the course of PD (8). NMS are valuable for the differential diagnosis of PD from atypical parkinsonisms (9) and may precede motor symptoms and influence patients’ quality of life (10). For example, mild cognitive impairment is associated with poor gait performance in patients with PD (11). Both motor and NMS vary among patients with PD and show complex interactions with each other.

Despite the substantial importance of clinical heterogeneity in PD, its underlying pathophysiology largely remains unclear. Numerous extrastriatal pathologies have been recently described as correlates for NMS. While the severity of dopaminergic depletion in different subtypes has been evaluated (2), the pathogenic changes in other non-dopamine systems are not well understood. Progressive degeneration of the cholinergic system has been widely implicated in PD (12). Besides its role in cognitive deterioration, this form of degeneration has been also proposed to be the functional link between cholinergic alterations and PD motor symptoms, sleep abnormalities, autonomic dysfunction, and altered olfactory function (12, 13). Cholinergic system-related NMS also affect PD motor classification and its stability (14, 15). Therefore, we speculated that changes in the cholinergic system, most importantly the nucleus basalis magnocellularis (NBM) and the pedunculopontine nucleus (PPN), may be associated with motor impairment and may influence the motor classification.

Diffusion tensor imaging (DTI) is a sensitive method for isolating white matter (WM) trajectories and exploring microstructural integrity in PD (16). DTI models each voxel as a single compartment. Free-water (FW) DTI uses an advanced 2-compartment diffusion model: one from the tissue and the other from free diffusing water (17); recent advancements in this technique have been shown to minimize the partial-volume effects of the cerebrospinal fluid and reflect neuroinflammation (18). In one study, FW imaging findings for the substantia nigra in different stages of PD were evaluated and were found to be a useful disease-state biomarker (16). FW imaging has also been shown to be informative for assessment of the cholinergic nucleus and cholinergic projection system (19–21), and has been shown to be a useful biomarker of cognitive symptoms in neurodegenerative disease. In the current study, we used cholinergic FW imaging as well as clinical data from the PPMI cohort of patients with idiopathic *de novo* PD to evaluate (1) the clinical and cholinergic FW imaging data in relation to the PD motor subtype at baseline and at the 4-year follow-up; (2) the correlation of cholinergic microstructural integrity and motor subscores; and (3) the ability of FW markers to detect changes in the PD phenotype after 4 years.

## Materials and methods

### Study sample

All the clinical and imaging data used in the study were downloaded from the PPMI database^1^ in August 2023. The PPMI is a comprehensive longitudinal, international, multicenter database consisting of clinical, genetic, neuroimaging and blood/cerebrospinal fluid (CSF) biomarker data of patients with early PD.

The inclusion criteria for the PD cohort in PPMI were as follows: idiopathic PD, early-stage disease classified as Hoehn–Yahr stage 1–2 at baseline, T1 imaging and DTI data available at baseline, and absence of anti-PD or anticholinergic medication. Each PPMI site received approval from its relevant institutional review board, and all participants provided written informed consent before participating in the project.

### Clinical evaluation and subtype classification

All baseline clinical evaluations were performed without dopaminergic replacement therapy, and 4-year follow-up evaluations were performed in the off-medication state. The levodopa-equivalent daily dose (LEDD; mg/day) was calculated from the dosages of the prescribed dopaminergic drugs using the standard conversion table (22). Motor dysfunction and disease severity were assessed on the basis of the UPDRS part II and III scores and the modified Hoehn and Yahr (H-Y) stages, respectively. For each patient, we obtained the rigidity subscore (total score of item 3.3), tremor subscore (sum of the scores for items 2.10 and 3.15–3.18), and PIGD score (sum of the scores for items 2.12, 2.13, and 3.10–3.12) from the PPMI. The TD/PIGD phenotype of PD classification was based on the mean tremor subscore/mean PIGD score ratio. If the ratio was >1.15 or the PIGD score was 0 and tremor subscore was >0, then the participant was classified into the TD subtype; if the ratio was <0.9, then the participant was classified into the PIGD subtype; and if the ratio was 0.9–1.15 or if the tremor and PIGD scores were 0, then the participant was classified into the ID subtype (5). The PIGD and ID subtypes were together classified as the “non-tremor” type.

Additionally, evaluations using Montreal Cognitive Assessment (MoCA), Parkinson’s disease-Autonomic (SCOPA-AUT), Geriatric Depression Scale (Short Version), State–Trait Anxiety Inventory (STAI), Epworth Sleepiness Scale (ESS), and Rapid Eye Movement Scale (REM) were also performed at baseline and at the 4-year follow-up.

### Dopamine transporter imaging

Dopamine transporter (DAT) imaging using I^123^ Ioflupane single-photon emission computed tomography (SPECT) was acquired at PPMI imaging centers in accordance with the PPMI imaging protocol, and sent to the Institute for Neurodegenerative Disorders for processing and calculation. SPECT raw projection data were imported to a HERMES (Hermes Medical Solutions, Skeppsbron 44, 111 30 Stockholm, Sweden) system for iterative reconstruction, then transferred to the PMOD (PMOD Technologies, Zurich, Switzerland) for subsequent processing. After attenuation correction, Gaussian filtering, and alignment and identification of the regions of interest, the striatal binding ratio was calculated as the ratio of the values for the target and reference regions (see text footnote 1). We acquired the data for the mean caudate, putamen, and striatum uptake relative to the uptake of the occipital area for analysis.

### MRI protocol and processing

**Figure 1:**
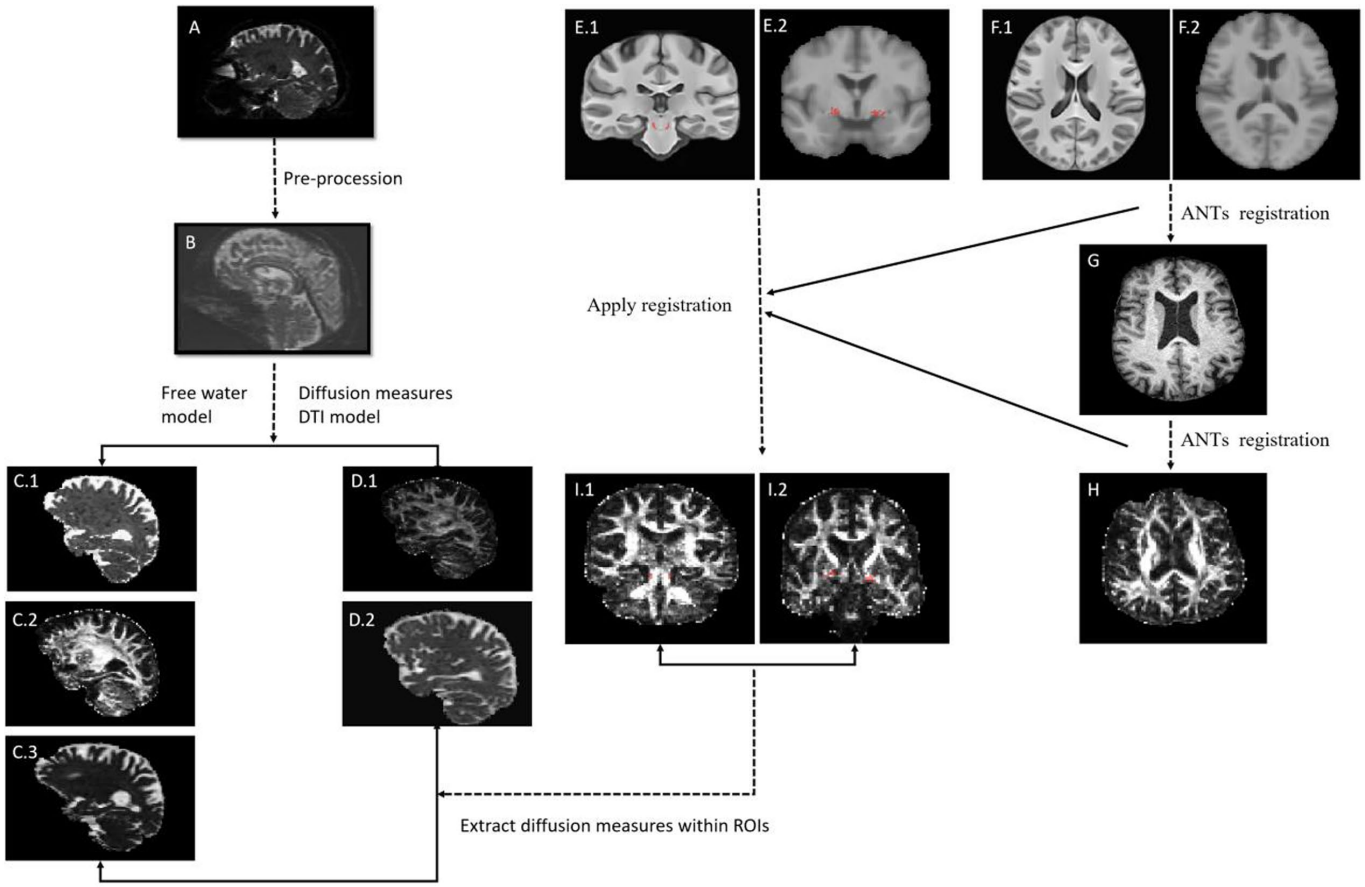
Overview of image processing. **(A)** Raw diffusion data; **(B)** Pre-processing diffusion data; **(C)** Free water (FW) related image, **(C.1)** FW image, **(C.2)** FW-corrected fractional anisotropy (FA) image, **(C.3)** FW-corrected mean diffusivity (MD) image; **(D)** Diffusion tensor imaging (DTI) related image, **(D.1)** FA image, **(D.2)** MD image; **(E.1)** PPN mask in standard space, **(E.2)** NBM mask in standard space; **(F.1)** Standard template where PPN mask are; **(F.2)** Standard template where NBM are; **(G)** Individual’s T1 image; **(H)** Individual’s FA image; **(I.1)** PPN mask in individual’s diffusion space, **(I.2)** NBM mask in individual’s diffusion mask. ANTs, Advanced Normalization Tools.

T1-weighted MRI and DTI were performed using Siemens 3 T scanners at multiple centers. The detailed MRI protocol is available in the PPMI database. We downloaded the baseline image data of the study participants. DTI data were preprocessed for denoising and removal of Gibbs artifacts and correction of head motion, eddy current-induced distortion, and intensity bias using the FMRIB Software Library (FSL). Multi-fiber directions within each voxel of the brain were estimated based on the ball-and-two-sticks model (23). With the processed images, fractional anisotropy (FA) and mean diffusivity (MD) images for each participant were obtained by using the “dtifit” function within FSL. FW, FW-corrected FA (FA-t), and FW-corrected MD (MD-t) values were derived from preprocessed DTI data using the two-compartment free-water elimination model (24). This model, which separates tissue-specific diffusion from the contribution of extracellular free water, was implemented using the Diffusion Imaging in Python (DIPY) package (version 1.4.0) (24, 25) with publicly available reference code^2^ (TensorImaging). The image processing procedure is summarized in Figure 1.

### Regions of interest

The NBM mask was based on the cholinergic nucleus 4 (Ch4) region of a cytoarchitectonic map of the cholinergic basal forebrain in the Montreal Neurological Institute (MNI) T1 space, which was derived by combining the findings of histological and MRI assessments of a postmortem brain (26). For the PPN, the mask was based on a PPN-specific high-resolution atlas, which was obtained by combining a recently constructed MNI-space unbiased quantitative susceptibility mapping atlas, a myelin staining histology human brain atlas, and an implicit representation-based self-supervised image super-resolution technique (27).

### Extraction of diffusivity metrics and volumetry for NBM and PPN

The mean FW, MD-t, and FA-t values were extracted from the NBM and the PPN. To ensure that these were constrained to gray matter (GM), the NBM was masked with the GM mask estimated from the segmentation of T1-weighted images (20). For the PPN, which had WM pathways from the brainstem running through it, the analysis was restricted to voxels with fractional anisotropy between 0.47 and 0.77, in accordance with previous studies (19, 20). To evaluate cell body damage in the cholinergic neurons, the NBM and PPN volumes in each individual’s diffusion space were calculated. The final NBM and PPN volumes were adjusted on the basis of the total intracranial volume [estimated using the Freesurfer’s SAMSEG segmentation (28)] to account for between-subject variability in head size.

### Statistical analysis

Statistical analysis was performed using Statistical Package for Social Sciences (SPSS 23.0) software. Continuous variables were expressed as mean ± standard deviation and compared using independent-samples *t*-test (normally distributed data) or the exact Mann–Whitney *U*-test (non-normally distributed data). Categorical variables were expressed as frequencies and compared using the chi-square test. Spearman’s correlation analysis was performed to evaluate the relationships between cholinergic imaging data and motor subscores at baseline. Multivariable binary logistic regression analysis was used to investigate the association of the baseline variables in the TD and non-TD subtypes and the stability of the subtypes at the 4-year follow-up. Zero-mean normalization was used for cholinergic imaging data in regression analysis.

## Results

### Clinical characteristics at baseline

A total of 150 patients were eligible for the baseline clinical evaluation. The baseline demographic and clinical data in the TD and non-TD groups are summarized in Table 1. The two groups showed significant differences in the years of education; UPDRS II, PIGD, and ESS scores; and the tremor subscore (*p* < 0.05). Although the TD group tended to show lower values on DAT imaging, significant intergroup differences were obtained only for the mean caudate (t = 2.143, *p* = 0.034). In a cross-sectional comparison of the baseline FW, FA-t, MD-t, and voxel values on *a priori* regions of the Ch4 and PPN between the TD and non-TD groups, none of these values for the regions of interest showed intergroup differences (Table 1).

**Table 1:** Demographic and clinical data in Parkinson’s disease patients with different subtypes at baseline and the 4-year follow-up.

Clinical variables	Baseline				4-year follow-up			
	Missing	TD group (*n* = 99)	Non-TD group (*n* = 51)	*p* value	Missing	TD group (*n* = 45)	Non-TD group (*n* = 39)	*p* value
Demographic features								
Male (*n*, %)	0/0	62 (62.63%)	34 (66.67%)	0.625	0/0	27 (60.0%)	30 (76.92%)	0.098
Age at visit (years)	0/0	61.38 ± 8.79	60.58 ± 10.87	0.795	0/0	65.44 ± 8.56	63.31 ± 10.45	0.306
Age at onset (years)	1/2	59.04 ± 9.02	59.19 ± 11.03	0.936	0/0	59.58 ± 9.12	57.55 ± 10.42	0.346
Education (years)	0/0	15.93 ± 2.77	14.24 ± 3.03	**0.002**	0/0	15.22 ± 3.28	15.51 ± 2.79	0.666
PD duration (months)	0/0	6.98 ± 7.21	6.20 ± 6.07	0.702	0/0	55.38 ± 6.30	54.82 ± 8.41	0.730
LEDD	-	-	-	-	0/0	516.55 ± 319.24	536.74 ± 285.30	0.762
Clinical characteristics								
Hoehn & Yahr	0/0	1.54 ± 0.50	1.65 ± 0.48	0.318	1/1	1.71 ± 0.51	2.03 ± 0.58	**0.010**
UPDRS II score	0/0	4.85 ± 3.66	6.65 ± 4.40	**0.009**	0/0	7.64 ± 4.56	9.38 ± 5.54	0.188
UPDRS III score	0/0	21.11 ± 9.18	19.49 ± 8.68	0.299	0/1	28.64 ± 10.86	29.08 ± 15.42	0.714
UPDRS total score	0/0	30.81 ± 13.10	32.08 ± 14.08	0.584	0/1	44.20 ± 15.54	46.82 ± 21.75	0.830
Rigidity subscore	0/0	4.00 ± 2.69	4.53 ± 2.91	0.268	0/0	5.36 ± 2.98	6.95 ± 3.52	**0.028**
Tremor subscore	0/0	6.43 ± 3.19	1.92 ± 2.05	**<0.001**	1/0	7.56 ± 3.08	2.10 ± 2.47	**<0.001**
PIGD subscore	0/0	0.75 ± 0.83	1.88 ± 1.36	**<0.001**	1/0	1.82 ± 1.57	4.31 ± 5.01	**0.026**
Non-motor symptoms								
MoCA	0/0	27.62 ± 2.04	27.39 ± 2.23	0.538	0/1	26.98 ± 2.58	27.68 ± 2.53	0.213
ESS	0/0	5.41 ± 3.17	6.88 ± 3.32	**0.009**	0/0	7.11 ± 4.39	9.13 ± 4.71	**0.046**
GDS	0/0	2.08 ± 2.36	2.90 ± 2.83	0.061	0/0	2.18 ± 2.54	2.59 ± 2.11	0.426
SCOPA-AUT	2/1	9.00 ± 6.47	8.88 ± 4.65	0.907	0/0	11.18 ± 5.88	13.18 ± 6.55	0.144
STAI	0/0	65.24 ± 19.17	68.27 ± 15.45	0.330	0/0	63.47 ± 17.63	65.44 ± 14.99	0.586
REM	0/0	3.89 ± 2.51	4.33 ± 2.59	0.333	1/0	4.05 ± 2.64	5.87 ± 3.66	**0.025**
DAT imaging								
Mean caudate score	0/0	1.94 ± 0.51	1.75 ± 0.51	**0.034**	8/5	1.55 ± 0.44	1.38 ± 0.42	0.114
Mean putamen score	0/0	0.81 ± 0.24	0.77 ± 0.29	0.441	8/5	0.57 ± 0.18	0.54 ± 0.21	0.216
Mean striatum score	0/0	1.37 ± 0.36	1.26 ± 0.38	0.079	8/5	1.06 ± 0.29	0.96 ± 0.29	0.161
Cholinergic imaging								
Ch4 volume (mm^3^)	0/0	416.27 ± 72.07	424.01 ± 0.41	0.566	0/0	439.43 ± 64.12	422.56 ± 68.87	0.249
FA-t-Ch4	0/0	0.50 ± 0.60	0.50 ± 0.64	0.771	0/0	0.50 ± 0.56	0.50 ± 0.67	0.559
MD-t-Ch4 *10^−3^	0/0	0.63 ± 0.14	0.60 ± 0.10	0.096	0/0	0.65 ± 0.16	0.58 ± 0.09	**0.029**
FW-Ch4	0/0	0.37 ± 0.05	0.37 ± 0.04	0.105	0/0	0.38 ± 0.51	0.36 ± 0.33	**0.042**
PPN volume (mm^3^)	0/0	65.94 ± 22.50	71.15 ± 25.33	0.200	0/0	68.84 ± 23.05	68.34 ± 20.50	0.918
FA-t-PPN	0/0	0.89 ± 0.37	0.90 ± 0.34	0.367	0/0	0.89 ± 0.04	0.90 ± 0.27	0.344
MD-t-PPN*10^−3^	0/0	0.36 ± 0.04	0.35 ± 0.05	0.906	0/0	0.36 ± 0.04	0.36 ± 0.04	0.557
FW-PPN	0/0	0.36 ± 0.28	0.36 ± 0.04	0.539	0/0	0.35 ± 0.30	0.36 ± 0.02	0.520

Results are expressed as mean ± standard deviation for continuous variables and as frequencies for categorical variables. Bold font indicates significant differences (*p* < 0.05).

TD, tremor-dominant subtype; LEDD, levodopa-equivalent daily dose; UPDRS, Unified Parkinson’s Disease Rating Scale; PIGD, postural instability and gait difficulty; MoCA, Montreal Cognitive Assessment; ESS, Epworth Sleepiness Scale; SCOPA-AUT, Parkinson’s disease-Autonomic; GDS, Geriatric Depression Scale; STAI, State–Trait Anxiety Inventory; REM, Rapid Eye Movement scale; DAT, dopamine transporter; Ch4, cholinergic nucleus 4; FA-t-Ch4, free-water-corrected fractional anisotropy in Ch4; MD-t-Ch4, free-water-corrected mean diffusivity in Ch4; FW-Ch4, free-water value for Ch4; PPN, pedunculopontine nucleus; FA-t-PPN, free-water-corrected fractional anisotropy in PPN; MD-t-PPN, free-water-corrected mean diffusivity in PPN; FW-PPN, free-water value for PPN.

### Correlation of cholinergic FW imaging findings and motor symptoms

**Figure 2:**
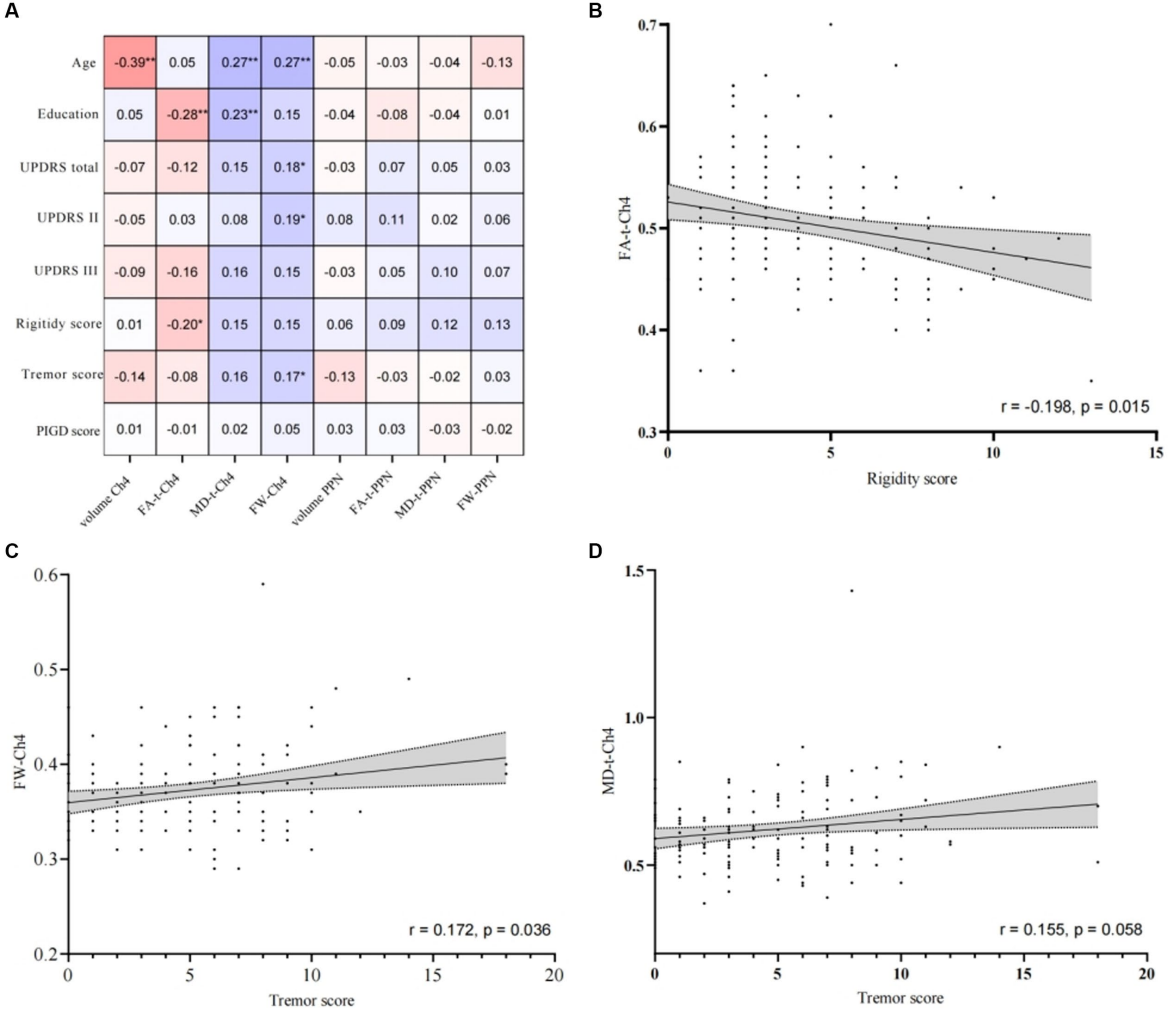
Spearman’s correlation analysis of cholinergic free-water imaging data and clinical data. **(A)** The correlation coefficients are shown in the table. **indicates *p* < 0.01 and *indicates *p* < 0.05. **(B)** Correlation between rigidity score and FW-corrected fractional anisotropy of cholinergic nucleus 4 (FA-t-Ch4); **(C)** correlation between the tremor subscore and the free-water value for cholinergic nucleus 4 (FW-Ch4); **(D)** correlation between the tremor subscore and the FW-corrected mean diffusivity of cholinergic nucleus 4 (MD-t-Ch4).

Analysis of the correlation between the findings of cholinergic FW imaging and clinical characteristics was performed in all PD patients (Figure 2A). In brief, age was negatively correlated with the volume of Ch4 and positively correlated with the FW value for Ch4 (FW-Ch4) and MD-t-Ch4. Years of education was negatively correlated with FA-t-Ch4 and positively correlated with MD-t-Ch4. FW-Ch4 was correlated with the UPDRS II and UPDRS total scores and the tremor subscore, while FA-t-Ch4 was negatively correlated with the rigidity subscore (Figures 2B–D). Our findings also showed trends for correlations between the UPDRS III score and FA-t-Ch4 (*r* = −0.161, *p* = 0.050) and MD-t-Ch4 (*r* = 0.155, *p* = 0.057) and between the tremor subscore and MD-t-Ch4 (*r* = 0.155, *p* = 0.058). Nevertheless, no correlation was observed between any of the clinical signs and the results of PPN diffusion tensor water imaging.

### Stability and fluctuation of PD subtypes

**Figure 3:**
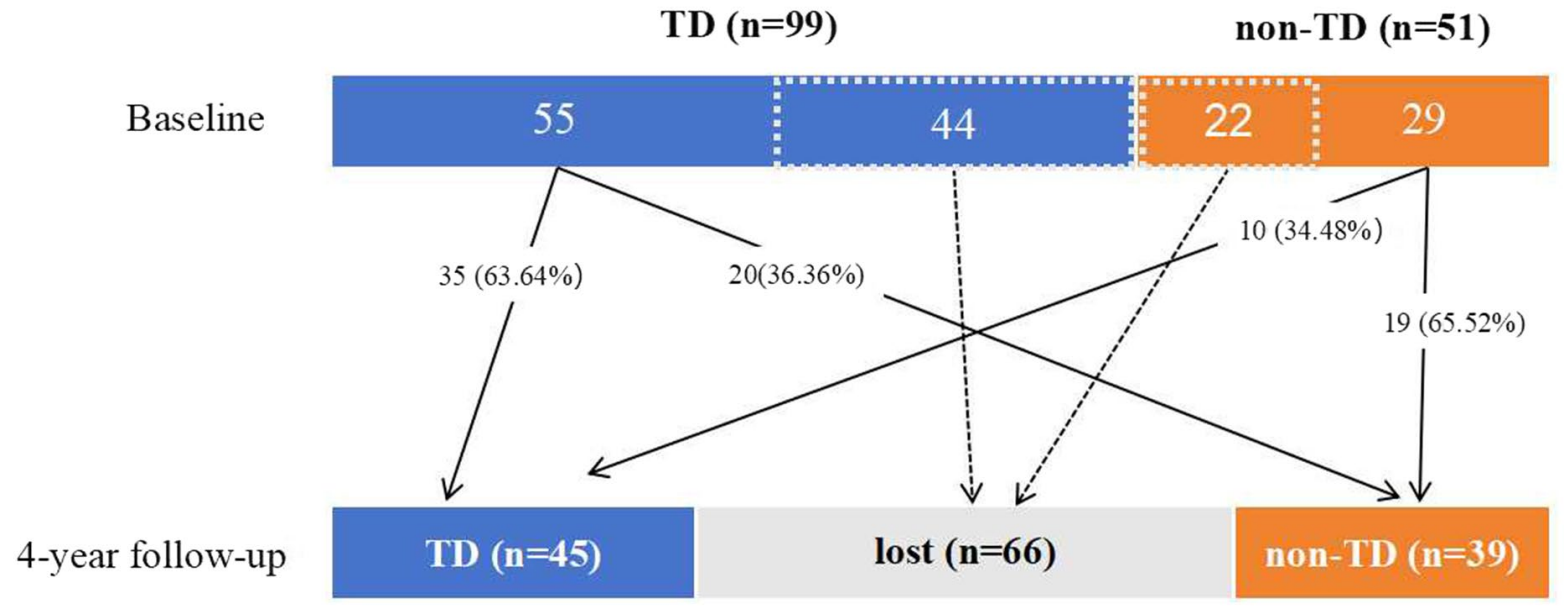
Fluctuations in Parkinson’s disease subtypes over the 4-year follow-up period.

Data for the stability and fluctuation of PD subtypes after 4 years are presented in Figure 3. The 4-year follow-up assessments were conducted in 84 (56.00%) individuals, and the remaining 66 (44.00%) individuals were excluded because of the lack of off-stage follow-up data. Among the 84 PD patients, 55 (65.48%) and 29 (34.52%) patients were categorized into the TD and non-TD (indeterminate, 13; PIGD, 16) types, respectively, at baseline. At the 4-year follow-up, the number of patients categorized into the TD and non-TD types was 45 (53.57%) and 39 (46.43%; indeterminate, 14 and PIGD, 25), respectively. Thus, 36.36% (20/55) of the cases initially classified under the TD subtype changed to the non-TD subtype, while 34.48% (10/29) of the cases initially classified under the non-TD subtype changed to the TD subtype.

### Association of cholinergic FW imaging data with PD subtypes at the 4-year follow-up

The clinical characteristics of patients categorized by the 4-year follow-up data are shown in Table 1. The H-Y stage and ESS and REM scores indicated greater severity in the non-TD group. However, other characteristics, including DAT imaging findings, showed no significant differences. Additionally, MD-t-Ch4 and FW-Ch4 were significantly higher in the TD group.

**Figure 4:**
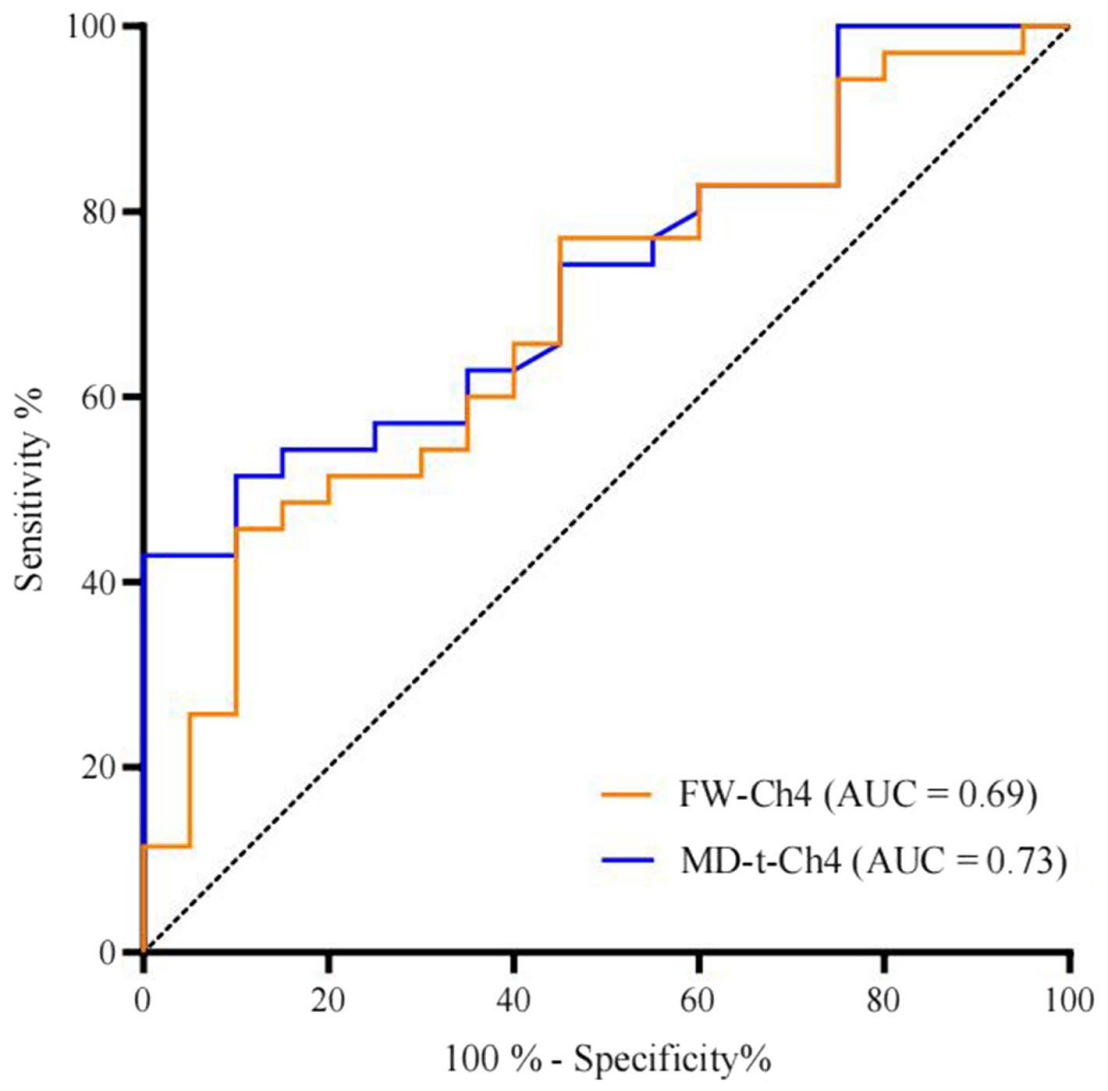
Receiver operating characteristic curves of FW imaging data for cholinergic nucleus 4 in relation to subtype stability over 4 years in the initial tremor-dominant subtype group.

Multivariate binary logistic regression analysis showed that normalized FW-Ch4 (nFW-Ch4) was a predictor of subtype at the 4-year follow-up. In the initial TD subgroup, both nFW-Ch4 and normalized MD-t-Ch4 (nMD-t-Ch4) could predict subtype stability separately; however, in the initial non-TD group, nMD-t-Ch4 was a predictor for subtype stability in model 3 but not in model 2 (Table 2). Receiver operating characteristic curve analysis showed that the initial nFW-Ch4 (OR: 0.304, 95% CI: 0.116–0.796, *p* = 0.015) and nMD-t-Ch4 (OR: 0.154, 95% CI: 0.035–0.672, *p* = 0.013) could adequately predict subtype stability at the 4-year follow-up in the initial TD group (Figure 4).

**Table 2:** Ch4 FW imaging data as a predictor of Parkinson’s disease subtype stability at the 4-year follow-up.

	Total sample				Initial TD group				Initial non-TD group			
	B	OR	95% CI	*p* value	B	OR	95% CI	*p* value	B	OR	95% CI	*p* value
Model 1 based on FW-Ch4												
Age	−0.05	0.95	0.90–1.01	0.080	−0.88	0.92	0.84–1.00	0.060	−0.05	0.95	0.86–1.04	0.273
Male	0.65	1.91	0.63–5.79	0.255	1.94	6.97	1.00–48.82	0.051	−0.09	0.92	0.12–6.88	0.932
REM	0.14	1.15	0.95–1.38	0.155	0.29	1.33	1.01–1.76	**0.042**	−0.03	0.97	0.69–1.35	0.850
ESS	0.02	1.02	0.90–1.17	0.729	−0.04	0.96	0.79–1.17	0.695	−0.02	0.98	0.79–1.21	0.856
H-Y	1.19	3.29	1.14–9.53	**0.028**	1.75	5.75	0.51–64.36	0.156	1.21	3.34	0.65–17.07	0.147
nFW-Ch4	−0.64	0.53	0.29–0.98	**0.041**	−1.19	0.30	0.12–0.80	**0.015**	0.04	1.04	0.24–4.42	0.963
Model 2 based on MD-t-Ch4												
Age	−0.05	0.95	0.90–1.01	0.102	−0.07	0.93	0.85–1.02	0.139	−0.08	0.93	0.84–1.02	0.129
Male	0.56	1.74	0.58–5.22	0.321	2.02	7.51	1.00–56.37	0.050	0.68	1.98	0.21–18.68	0.552
REM	0.14	1.15	0.95–1.38	0.151	0.28	1.33	0.99–1.78	0.063	−0.08	0.92	0.65–1.31	0.645
ESS	0.03	1.10	0.91–1.18	0.644	0.04	1.04	0.84–1.29	0.731	0.03	1.03	0.82–1.28	0.817
H-Y	1.04	0.95	0.90–1.01	**0.049**	1.01	2.75	0.23–33.58	0.428	1.14	3.13	0.68–14.55	0.145
nMD-t-Ch4	−0.60	0.55	0.29–1.03	0.060	−1.88	0.15	0.04–0.67	**0.013**	1.18	3.25	0.60–17.63	0.171
Model 3 based on FW-Ch4 & MD-t-Ch4												
Age	−0.05	0.95	0.90–1.01	0.089	−0.07	0.93	0.85–1.03	0.158	−0.07	0.93	0.82–1.06	0.274
Male	0.63	1.88	0.62–5.73	0.267	2.00	7.39	0.96–57.05	0.055	0.38	1.45	0.07–32.77	0.814
REM	0.14	1.15	0.95–1.38	0.153	0.28	1.32	0.98–1.79	0.068	−0.64	0.53	0.25–1.11	0.090
ESS	0.03	1.03	0.90–1.17	0.710	0.41	1.04	0.83–1.31	0.721	0.07	1.07	0.80–1.43	0.652
H-Y	1.16	3.18	1.08–9.36	**0.036**	0.99	2.70	0.21–33.97	0.443	0.45	1.56	0.97–2.61	0.067^#^
nFW -Ch4	−0.52	0.60	0.20–1.82	0.365	0.076	1.08	0.23–5.12	0.924	−0.75	0.47	0.22–1.02	0.055^#^
nMD-t-Ch4	−0.15	0.86	0.27–2.72	0.801	−1.95	0.14	0.02–1.20	0.073	0.76	2.17	1.07–4.41	**0.032** ^#^

TD, tremor-dominant subtype; ESS, Epworth Sleepiness Scale; REM, Rapid Eye Movement scale; Ch4, cholinergic nucleus 4; nFA-t-Ch4, normalized free-water-corrected fractional anisotropy in Ch4; nMD-t-Ch4:normalized free-water-corrected mean diffusivity in Ch4; FW, free-water value for Ch4; H-Y, Hoehn & Yahr.

^#^ the value of H-Y, nFW -Ch4 and nMD-t-Ch4 were magnified by a factor of 10. Bold font indicates significant differences (*p* < 0.05).

## Discussion

In this study, we used FW imaging to investigate the microstructural changes of the two main cholinergic nuclei in different PD subtypes and determine their associations with motor function. Our findings revealed that FW-Ch4 was associated with the tremor subscore, while FA-t-Ch4 was negatively associated with the rigidity subscore. Moreover, nFW-Ch4 showed discriminative ability for predicting the stability of PD subtypes over 4 years. However, we did not observe any relationship between the volume of the cholinergic nuclei and motor symptoms, indicating that volume changes were not significant in the early stage and that the microstructural diffusion alterations measured by FW imaging may be more sensitive markers. These findings also indicated that in comparison with PPN, Ch4 is more associated with the motor score and motor subtypes.

The cholinergic nuclei NBM and PPN are at the epicenter of the cholinergic system, with most cholinergic projections in the brain originating from them and connecting with the brainstem, thalamus, and cortex (29). The cholinergic system has been associated with motor and NMS in PD (12, 13). Because of the interplay between cognitive and gait functions (30), previous studies mainly focused on the association of the cholinergic system with gait problems, freezing of gait, falls, and postural control (31, 32). DTI studies indicated that baseline increased axial diffusivity of the PPN detected with DTI was independently associated with the development of PIGD symptoms (33), and that FW in the cholinergic basal forebrain was correlated with a worse PIGD score, which may be mediated by attentional function (21). In addition to the cholinergic nuclei, the integrity of the cholinergic pathway also plays a role in motor symptoms (21). However, our study did not identify a significant relationship between the FW imaging data for the cholinergic nucleus and PIGD severity, and our findings could not characterize the role of the PPN. This may have occurred since the patients in the present study had early-stage PD. Thus, follow-up evaluation of the changes in cholinergic FW imaging may provide a better understanding of this aspect.

Surprisingly, we found that increased FW was positively correlated with the tremor subscore, which indicated that tremor severity was associated with microchanges in the Ch4 in the early *de novo* stage of PD. Tremor is one of the most prevalent symptoms in PD, but the pathophysiology of parkinsonian tremors remains poorly understood (34). Postmortem studies have demonstrated that PD patients with the TD subtype had less cell loss in the dopaminergic and nondopaminergic neural circuits than those without tremors (35). Moreover, the severity of parkinsonian tremors has been reported to be unrelated to the degree of dopaminergic denervation (36), consistent with our findings showing that the TD group had higher levels of dopamine in the caudate nucleus than the non-TD group. A growing body of evidence suggests that parkinsonian tremors may be related to degeneration of non-dopaminergic systems such as the serotoninergic and noradrenergic systems (34). Few studies have linked the cholinergic system to the severity of tremors. In fact elevated cholinergic activity is an important pathological marker in early PD, and anticholinergic therapy has shown remarkable effects on tremor symptoms. One study also indicated that cholinergic relevant functional reactivity is associated with the dopamine responsiveness of tremors in PD (37). In our study, an increased FW indicated degeneration or inflammation of Ch4, although the correlation between the FW-Ch4 alterations and acetylcholine release was not clear. Nevertheless, these findings provide the basis for further research on the role of cholinergic system damage in PD tremors.

Rigidity is another cardinal motor sign in PD, but it has been evaluated in only a relatively small number of clinical and experimental studies. An overnight sleep study showed higher and more symmetric upper limb rigidity indexes during wakefulness in patients with PD and REM sleep without atonia (RSWA) than in patients without RSWA and healthy controls (38). Although no correlation was observed between the rigidity subscore and the REM score in our sample, the findings could not clarify whether these scores are connected by a cholinergic mechanism.

Many studies on motor subtypes have been sourced from the PPMI database (2, 6, 7, 39), and the differences in other indicators, such as CSF biomarkers, among different subtypes were not shown in this study. In this study, the majority of the patients belonged to the TD subtype at baseline, while 35.71% of the individuals showed a shift in the motor subtypes at the fourth-year visit. Fluctuations in PD motor subtypes are common (7, 40, 41), especially during the first 2 years (42). These subtype shifts may be related to the progression of PIGD (40, 41), the effects of NMS (14), and drug interventions (41). In this regard, “true” TD patients are thought to show better subtype stability over time (40). The importance of non-motor manifestations as drivers of PD subtyping and prognosis has been proposed. The type composite with critical non-motor features (named as “diffuse malignant” type) demonstrates a more profound dopaminergic deficit, increased atrophy in PD brain networks, a more Alzheimer’s disease-like CSF profile, and faster progression of motor and cognitive deficits (2). The pathophysiology of these NMS, especially cognitive impairment and rapid eye movement sleep behavior disorder, are related to alterations of the cholinergic systems (12), indicating the role of the cholinergic system in the motor subtype. Meanwhile, as the disease progresses, resulting in a more severe dopaminergic deficit, the effect of the cholinergic system on subtype classification may increase. In this study, TD patients had higher putamen dopamine transporter availability than non-TD patients at baseline, but not at the 4-year follow-up. Moreover, the asymmetry in striatal denervation indices between the TD and PIGD subtypes has been reported to be the most prominent at the baseline, but this difference became non-significant after the 4-year follow-up (43). As the effects of dopamine reduce, the effects of the cholinergic system may become more apparent.

Patients showing PD with PIGD often face a more malignant prognosis than patients with TD. A model capable of identifying potential PIGD converters from the TD group would enable more personalized and potentially more effective therapeutic interventions. In our studies, the initial nFW-Ch4 and nMD-t-Ch4 showed predictive value for the 4-year motor subtype in the TD subgroup. FW indicates degeneration or inflammation, and MD is associated with alterations due to changes in membrane integrity. These changes may help us understand the pathological processes underlying the motor subtype in PD. Further studies in larger populations are required to validate these findings.

### Limitations

This study had some limitations that require consideration. First, the sample size of this study was relatively small, and some cases were lost during follow-up. Second, we did not consider NMS in our subtyping algorithm and chose the traditional scoring method for grouping motor subtypes, which may have more widespread application to clinical care. However, PD motor subtypes determined by different established algorithms are inconsistent and unstable over time (40), and different categorizations may yield different outcomes. Third, to reduce the interference of medication, we chose to evaluate patients in the off state. However, in the PPMI study protocol, the off state only refers to overnight withdrawal of the patients’ anti-parkinsonian medications. Consequently, the drugs may not really have been metabolized to completion. Fourth, the majority of DTI data in the PPMI database were acquired using a single-shell protocol, which generally yields lower-quality FW analysis than multiple-shell DTI (17). Moreover, PPMI is a multicenter database, and we did not distinguish or correct the data from different centers. Fifth, we did not investigate the changes in cholinergic FW imaging data over time, which can precisely reflect the mechanisms underlying the disease-induced changes. Moreover, FW imaging only provided an indirect measure, and could not directly exhibit the neurotransmitter-related alterations in brain.

## Conclusion

Our findings indicate that motor symptoms in PD were correlated with FW imaging data for the cholinergic nucleus and that nFW-Ch4 can be used to predict the transition of motor subtypes over a 4-year follow-up period, especially in patients initially classified into the TD group. Thus, our study provides valuable insights regarding the association of motor subtypes with the cholinergic system.

## Data Availability

The original contributions presented in the study are included in the article/supplementary material, further inquiries can be directed to the corresponding authors.

## Footnotes

^1^ http://www.ppmi-info.org

^2^ https://github.com/sameerd/DiffusionTensorImaging
